# Peptide-Induced Amyloid-Like Conformational Transitions in Proteins

**DOI:** 10.1155/2015/723186

**Published:** 2015-09-08

**Authors:** Vladimir Egorov, Natalia Grudinina, Andrey Vasin, Dmitry Lebedev

**Affiliations:** ^1^FSBI Research Institute of Influenza, Ministry of Health of the Russian Federation, 15/17 Professor Popova Street, Saint-Petersburg 197376, Russia; ^2^FSBI Petersburg Nuclear Physics Institute, NRC Kurchatov Institute, Orlova Roscha, Gatchina 188300, Russia; ^3^FSBSI Institute of Experimental Medicine, 12 Akademika Pavlova, Saint-Petersburg 197376, Russia; ^4^FSBI Federal Almazov Medical Research Centre, 2 Akkuratova Street, St. Saint-Petersburg 197341, Russia; ^5^Saint-Petersburg State Polytechnical University, 29 Polytechnicheskaya Street, Saint-Petersburg 195251, Russia

## Abstract

Changes in protein conformation can occur both as part of normal protein functioning and during disease pathogenesis. The most common conformational diseases are amyloidoses. Sometimes the development of a number of diseases which are not traditionally related to amyloidoses is associated with amyloid-like conformational transitions of proteins. Also, amyloid-like aggregates take part in normal physiological processes such as memorization and cell signaling. Several primary structural features of a protein are involved in conformational transitions. Also the protein proteolytic fragments can cause the conformational transitions in the protein. Short peptides which could be produced during the protein life cycle or which are encoded by short open reading frames can affect the protein conformation and function.

## 1. Conformational Diseases

**Figure 1:**
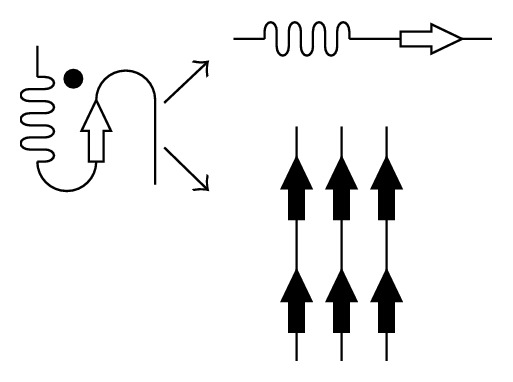
Scheme of protein misfolding leading to either loss of function (upper right) or appearing of the new properties (lower right). The normal, functioning protein is indicated with a black dot.

Conformational diseases are caused by changes in protein tertiary structures and the associated loss of existing functions or the appearing of the new properties such as oligomerization ability. The most common conformational diseases are amyloidoses which arise when proteins, having lost their native tertiary structure, acquire the ability to form oligomers toxic to cells, as well as insoluble fibrils resistant to proteolysis (Figure 1).

Currently, more than 30 human diseases associated with fibril formation in organs and tissues have been discovered. Some of them are associated with hereditary factors such as mutations in protein-encoded gene, for example, transthyretin amyloidosis (ATTR); some of them are caused by changes in protein environment, for example, beta-2-microglobulin amyloidosis (Ab2M) [1]. The most common types of amyloidosis include Alzheimer's disease [2], which is a localized amyloidosis and develops primarily among the elderly, as well as immunoglobulin light chains amyloidosis, which are caused by chronic pathological processes [3].

## 2. Amyloidosis-Like Diseases

It should be noted that although not all toxic oligomers are prefibrillar in nature, they still may possess structural similarities with prefibrillar oligomers. Their formation can accompany the development of a number of diseases not traditionally related to amyloidosis due to a lack of specific morphological traits in the analysis of the affected tissues. The processes leading to the development of amyloidosis may also, in a less severe manner, participate in the pathogenesis of other diseases. For example, the formation of beta-structured oligomers capable of inducing apoptosis via activation of certain intracellular mechanisms, such as changing mitochondrial permeability and releasing cytochrome C into the cytoplasm, is characteristic of the influenza virus A PB1-F2 protein [4] as well as mutant forms of myocilin [5] which participate in the pathogenesis of select familial forms of open-angle glaucoma. It has been shown that the tetramerization domain of the p53 protein is capable of forming amyloid fibrils. In this case, the presence of the mutation R337H, associated with the development of adrenocortical carcinomas, stabilizes the beta-conformation in p53 oligomers. Reversible fibrillogenesis of the protein is preceded by the loss of native tertiary structure, along with the reorganization and formation of intermolecular beta-structures [6].

## 3. Amyloid-Like Oligomers as Part of the Normal Functioning of Proteins

There is increasing evidence indicating a role for amyloid-like aggregates in physiological processes, such as memorization and cell signaling. APP knockout mice demonstrate a reduced capacity for memorization, and animal behavioral studies have shown that, by all accounts, Amyloid Precursor Protein (APP) and its proteolytic fragments play a role in long-term memory formation in adulthood [7]. The role of amyloid-like aggregates in the process of memorization has also been described for* Drosophila* [8]. Amyloid-like protein MAVS oligomerization is necessary for the formation of acellular response to viral infection [9]. The proteins RIP1 and RIP3, components of the TNF-induced apoptotic pathway, have been shown to also cause necroptosis through the formation of stable heterooligomeric amyloidal signaling complexes when the caspases responsible for the cleaving of RIP1 and RIP3 are inhibited, such as during certain viral infections [10]. Thus, it is clear that conformational changes in proteins and formation of amyloid oligomers play a role not only in the pathogenesis of amyloidosis-like diseases, but also in the normal operations for a number of proteins.

## 4. The Role of Primary Structure Motifs in Conformational Transitions

Here we will consider several primary structural features capable of causing conformational transitions in proteins. It is important to note that, despite the differences in the native structure among fibrillogenic proteins, the protein tertiary structures formed while transitioning to a beta-conformation during fibrillogenesis nevertheless share a number of common features [11]. The ability for a large number of proteins to form amyloid-like fibrils* in vitro* found its explanation in the work of Greenwald and Riek [12], where it was hypothesized that there exists a common origin for tertiary motifs able to form beta-structures typical of amyloid-like aggregates. In other words, a number of proteins over the course of evolution have retained a hidden capacity for oligomerization due to the formation of hydrogen bonds among former beta stretches in monomers. This means that, under certain environmental conditions, the native state of a protein containing stable alpha-helices can become destabilized, thereby allowing the transition to a beta-conformation protostate, which is stabilized due to intermolecular interactions [13].

The presence of alpha-helices and beta-sheets is often associated with a periodicity within a protein's primary structure [14]. It has been shown that the substitution of some amino acids in alpha-structured peptides could lead to a transition in the peptide to a beta-conformation and the formation of amyloid fibrils [15].

The simplest example of a commonly shared primary structural feature would be polyamino acid tracts. Some polyamino acids, such as polylysine, are able to exist in either an alpha- or beta-conformation. Moreover, it is noted that the longer the chain, the easier the transition to a beta-conformation [16]. Polyglutamine tracts play an important role in the conformational transitions of huntingtin [17], while insertion of certain non-Q residues into polyQ tracts within yeast prion proteins promotes fragmentation of said prions, thereby increasing the total number of prion particles and ensuring inheritance by the next generation [18]. The role of various polyamino acids in amyloid structure formation is considered in [19]. Another primary structure peculiarity among proteins capable of conformational transitions is the presence of amino acid repeats. The role of repeats in fibrillogenesis has been shown for the prion protein PrP [20] and for alpha-synuclein [21]. The leucine zipper motif located within myocilin usually forms an alpha-helix structure but is also prone to fibrillogenesis even under normal physiological conditions [22]. It would appear that the presence of easily identifiable repetitive sequences in these proteins is evidence of their relatively recent evolutionary origin [23].

**Figure 2:**
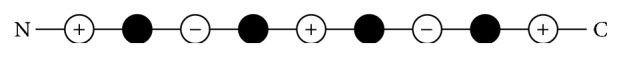
Scheme of an ionic self-complementary motif (iSCM). Positively charged amino acid residues are designated with a plus sign (+) and the negatively charged with a minus sign (−).

Yet another motif that can be found among proteins capable of conformational transitions is the ionic self-complementary motif (iSCM), which contains oppositely charged residues periodically arranged within the protein primary structure (Figure 2).

For a number of chaperone proteins, such as Hsp70 and MjHSP16.5, iSCMs play a significant role in the stabilization of secondary structures and interactions between the subunits [24]. Ionic self-complementary motifs in peptides have the following characteristics: they can exist both in a stable alpha-conformation, stabilized by intramolecular interactions, and in a beta-conformation, stabilized by intermolecular interactions. When in a beta-conformation, iSCMs are prone to amyloid fibril formation [25].

## 5. Induction of Conformational Transitions

**Figure 3:**
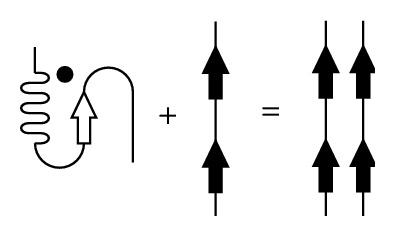
Scheme of a misfolded-protein-induced conformational transition. A normally folded protein (indicated by a black dot) interacts with a misfolded protein (black arrows), leading to the normally folded protein also adopting a misfolded conformation.

In the case of conformational diseases, an alpha-to-beta transition in the protein secondary structure may be induced by a change in external conditions, leading to the loss of native protein conformation, or by a process known as seeding, wherein other protein molecules already in an amyloidogenic conformation are responsible for inducing the conformation transition (Figure 3). It should be noted that during fibrillogenesis seeding in most cases is protein-specific [26].

**Figure 4:**
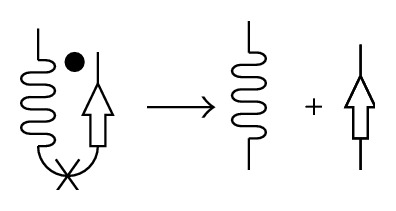
Scheme of a proteolytic-fragment-mediated conformational transition. Proteolytic cleavage and generation of the proteolytic fragment.

**Figure 5:**
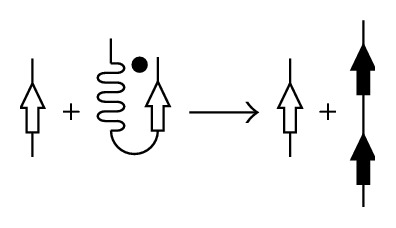
Scheme of a proteolytic-fragment-mediated conformational transition. Induction of the conformational transition for a whole protein by the proteolytic fragment.

The induction of conformational transitions can also be caused by amyloidogenic protein fragments (Figures 4 and 5). This has been specifically shown for the Syrian hamster prion protein PrP [27] and alpha-lactalbumin [28, 29]; it has also been shown that the NAGDVAFV peptide fibrils of lactoferrin are capable of inducing specific binding to the whole protein [30], while lysozyme fibrillogenesis is accelerated by the nicking or adding of fragments formed after its autohydrolysis by aspartate [31].

In some cases, short peptides are capable of not only facilitating the adoption of an amyloidogenic protein conformation in its parent protein, but also causing changes in the parent protein's functional secondary structures. For example, it has been shown that the antiviral activity of the PB1 (6-13) peptide of influenza virus PB1 protein affects the secondary structure of the N-terminal domain of the PB1 protein itself [32, 33].

**Figure 6:**
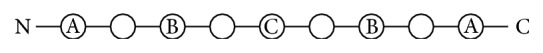
Scheme of a mirror-symmetrical motif (MSM). Identical amino acid residues are designated by the same letters.

It is interesting to note that those peptides for the prion protein PrP [27], alpha-lactalbumin [28, 29], and PB1 [32], all of which are capable of inducing a parent protein conformational transition, contain mirror-symmetrical motifs (MSMs) within their primary structures (Figure 6) [34]. Mirror-symmetrical motif formation can arise due to amplification of repeats in DNA and have a role in the formation of the tertiary structure in proteins [35].

Investigation into the influence of short peptides, including those containing MSM, iSCM, and amino acid repeats, upon protein conformation is necessary for the elucidation of protein metabolism mechanisms under normal and pathological conditions, as well as the role of short peptides in the stabilization of protein conformation. In addition, further research on how to detect short peptides encoded from short open reading frames as well as revealing their mechanisms of production is also needed [36].

## 6. Conclusion

Conformational changes play an important role both in disease pathology and in the normal functioning of proteins. Certain primary structural motifs, such as MSMs, iSCMs, and amino acid repeats, have been shown to be involved in such transitions, while proteolytic fragments can participate in the induction of conformational transitions in proteins not only for normal, biological purposes, but also due to pathological reasons. Finally, application of peptide analogues for proteolytic regulatory fragments could become a new approach for the development of drugs aimed at treatment of conformational diseases.

## References

[1] Sipe J. D., Benson M. D., Buxbaum J. N. (2014). Nomenclature 2014: amyloid fibril proteins and clinical classification of the amyloidosis. *Amyloid*.

[2] Castellani R. J., Rolston R. K., Smith M. A. (2010). Alzheimer disease. *Disease-a-Month*.

[3] Real de Asúa D., Costa R., Galván J. M., Filigheddu M. T., Trujillo D., Cadiñanos J. (2014). Systemic AA amyloidosis: epidemiology, diagnosis, and management. *Clinical Epidemiology*.

[4] Chevalier C., Al Bazzal A., Vidic J. (2010). PB1-F2 influenza A virus protein adopts a beta-sheet conformation and forms amyloid fibers in membrane environments. *The Journal of Biological Chemistry*.

[5] Orwig S. D., Perry C. W., Kim L. Y. (2012). Amyloid fibril formation by the glaucoma-associated olfactomedin domain of myocilin. *Journal of Molecular Biology*.

[6] Lee A. S., Galea C., DiGiammarino E. L. (2003). Reversible amyloid formation by the p53 tetramerization domain and a cancer-associated mutant. *Journal of Molecular Biology*.

[7] Senechal Y., Kelly P. H., Dev K. K. (2008). Amyloid precursor protein knockout mice show age-dependent deficits in passive avoidance learning. *Behavioural Brain Research*.

[8] Majumdar A., Cesario W. C., White-Grindley E. (2012). Critical role of amyloid-like oligomers of *Drosophila* Orb2 in the persistence of memory. *Cell*.

[9] Moresco E. M. Y., Vine D. L., Beutler B. (2011). Prion-like behavior of MAVS in RIG-I signaling. *Cell Research*.

[10] Li J., McQuade T., Siemer A. B. (2012). The RIP1/RIP3 necrosome forms a functional amyloid signaling complex required for programmed necrosis. *Cell*.

[11] Nelson R., Sawaya M. R., Balbirnie M. (2005). Structure of the cross-*β* spine of amyloid-like fibrils. *Nature*.

[12] Greenwald J., Riek R. (2012). On the possible amyloid origin of protein folds. *Journal of Molecular Biology*.

[13] Tzotzos S., Doig A. J. (2010). Amyloidogenic sequences in native protein structures. *Protein Science*.

[14] Xiong H., Buckwalter B. L., Shieh H.-M., Hecht M. H. (1995). Periodicity of polar and nonpolar amino acids is the major determinant of secondary structure in self-assembling oligomeric peptides. *Proceedings of the National Academy of Sciences of the United States of America*.

[15] Takahashi Y., Ueno A., Mihara H. (2000). Mutational analysis of designed peptides that undergo structural transition from alpha helix to beta sheet and amyloid fibril formation. *Structure*.

[16] Dzwolak W., Muraki T., Kato M., Taniguchi Y. (2004). Chain-length dependence of *α*-helix to *β*-sheet transition in polylysine: model of protein aggregation studied by temperature-tuned FTIR spectroscopy. *Biopolymers*.

[17] Kim M. (2013). Beta conformation of polyglutamine track revealed by a crystal structure of huntingtin N-terminal region with insertion of three histidine residues. *Prion*.

[18] Alexandrov A. I., Ter-Avanesyan M. D. (2013). Could yeast prion domains originate from polyQ/N tracts?. *Prion*.

[19] Fändrich M., Dobson C. M. (2002). The behaviour of polyamino acids reveals an inverse side chain effect in amyloid structure formation. *The EMBO Journal*.

[20] Dong J., Bloom J. D., Goncharov V. (2007). Probing the role of PrP repeats in conformational conversion and amyloid assembly of chimeric yeast prions. *The Journal of Biological Chemistry*.

[21] Tashiro M., Kojima M., Kihara H. (2008). Characterization of fibrillation process of *α*-synuclein at the initial stage. *Biochemical and Biophysical Research Communications*.

[22] Egorov V. V., Grudinina N. A., Lebedev D. V. (2013). Amyloidogenic peptide homologous to fragment 129–148 of human myocilin. *Prion*.

[23] Andrade M. A., Perez-Iratxeta C., Ponting C. P. (2001). Protein repeats: structures, functions, and evolution. *Journal of Structural Biology*.

[24] Farnsworth P. N., Singh K. (2000). Self-complementary motifs (SCM) in *α*-crystallin small heat shock proteins. *FEBS Letters*.

[25] Altman M., Lee P., Rich A., Zhang S. (2000). Conformational behavior of ionic self-complementary peptides. *Protein Science*.

[26] Krebs M. R. H., Morozova-Roche L. A., Daniel K., Robinson C. V., Dobson C. M. (2004). Observation of sequence specificity in the seeding of protein amyloid fibrils. *Protein Science*.

[27] Nguyen J., Baldwin M. A., Cohen F. E., Prusiner S. B. (1995). Prion protein peptides induce *α*-helix to *β*-sheet conformational transitions. *Biochemistry*.

[28] Egorov V. V., Solovyov K. V., Grudinina N. A. (2007). Atomic force microscopy study of peptides homologous to beta-domain of alpha-lactalbumins. *Protein and Peptide Letters*.

[29] Egorov V. V., Lebedev D. V., Shaldzhyan A. A. (2014). A conservative mutant of a proteolytic fragment produced during fibril formation enhances fibrillogenesis. *Prion*.

[30] Nilsson M. R., Dobson C. M. (2003). In vitro characterization of lactoferrin aggregation and amyloid formation. *Biochemistry*.

[31] Mishra R., Sörgjerd K., Nyström S., Nordigården A., Yu Y.-C., Hammarström P. (2007). Lysozyme amyloidogenesis is accelerated by specific nicking and fragmentation but decelerated by intact protein binding and conversion. *Journal of Molecular Biology*.

[32] Egorov V. V., Matusevich O. V., Shaldzhyan A. A. (2013). Structural features of the peptide homologous to 6–25 fragment of influenza A PB1 protein. *International Journal of Peptides*.

[33] Matusevich O. V., Egorov V. V., Gluzdikov I. A. (2015). Synthesis and antiviral activity of PB1 component of the influenza A RNA polymerase peptide fragments. *Antiviral Research*.

[34] Shpakov A. O. (2000). The mirror-type internal symmetry in the primary structure of proteins: detection and functional role (review). *Journal of Evolutionary Biochemistry and Physiology*.

[35] Pinotsis N., Wilmanns M. (2008). Protein assemblies with palindromic structure motifs. *Cellular and Molecular Life Sciences*.

[36] Andrews S. J., Rothnagel J. A. (2014). Emerging evidence for functional peptides encoded by short open reading frames. *Nature Reviews Genetics*.

